# Supplementary material for: Neuroinflammatory crosstalk between microglia and astrocytes increases viral replication in an iPSC-derived model of CNS HIV infection
Source: bioRxiv. 2025 Sep 4:2025.08.29.673049. Preprint. [Version 2] doi: 10.1101/2025.08.29.673049 (PMC12424981; doi:10.1101/2025.08.29.673049)

bioRxiv preprint doi: https://doi.org/10.1101/2025.08.29.673049; this version posted September 4, 2025. The copyright holder for this preprint (which was not certified by peer review) is the author/funder, who has granted bioRxiv a license to display the preprint in perpetuity. It is made available under aCC-BY 4.0 International license.

Supplementary Figure 1: (a). Expression of indicated HIV-entry related genes in iMg at 11 DIV compared to expression in MDM in log transformed transcripts per million (b). Heatmap indicating relative levels of indicated cytokines and chemokines produced by HIV-infected MDM (left) or iMg (middle left) compared to mock-infected iMg (middle right) or iMg treated with 100 ng/mL LPS (right) at the protein level. Data are condensed from 2(MDM) to 5 (iMg) biological replicates. **** = $p < 0.0001$. Multiple paired T-tests.

Supplementary Figure 2: (a). Expression of indicated TNFα receptor genes in MDM and iMg with or without infection with HIV Jago at 50 ng/mL in log transformed transcripts per million. **** = $p < 0.0001$. Multiple paired T-tests.

Supplementary Figure 3: (a). Raw pg/mL values measured by biorad Luminex of IL-6 produced by HIV-infected iMg monocultures (circles), HIV-infected iMg + iAst coclutures (triangles), or iAst treated with conditioned media from HIV-infected iMg (inverted triangles). Data include 3 (iAst) or 5 (iMg, CC) biological replicates. (b). TNFα levels measured by alphalisa in iMg monocultures or iMg/iAst cocultures treated with vehicle (PBS) or 100 ng LPS. Technical replicates within a group of biological replicates are indicated by distinct colors, and averages from a given biological replicate are expressed as the overlaid solid-colored symbols. Data include 2 biological replicates. * = $p < 0.05$, *** = $p< 0.001$. Multiple paired T-tests(a), One-Way ANOVA(b).

Supplementary Figure 4: (a). Manual counts of nucleated IBA1-positive microglia per tile-scan via widefield microscopy at 40X in the indicated culture conditions * = $p< 0.05$. Multiple paired T-tests.

Supplementary Figure 5: (a). Cathepsin B measured in culture supernatants from indicated groups at PID 12. Data are condensed from four biological replicates and plotted as fold-change vs the negative control group, iMg + Veh. * = $p < 0.05$, **** = $p < 0.0001$. Mann-Whitney test.

bioRxiv preprint doi: https://doi.org/10.1101/2025.08.29.673049; this version posted September 4, 2025. The copyright holder for this preprint (which was not certified by peer review) is the author/funder, who has granted bioRxiv a license to display the preprint in perpetuity. It is made available under aCC-BY 4.0 International license.

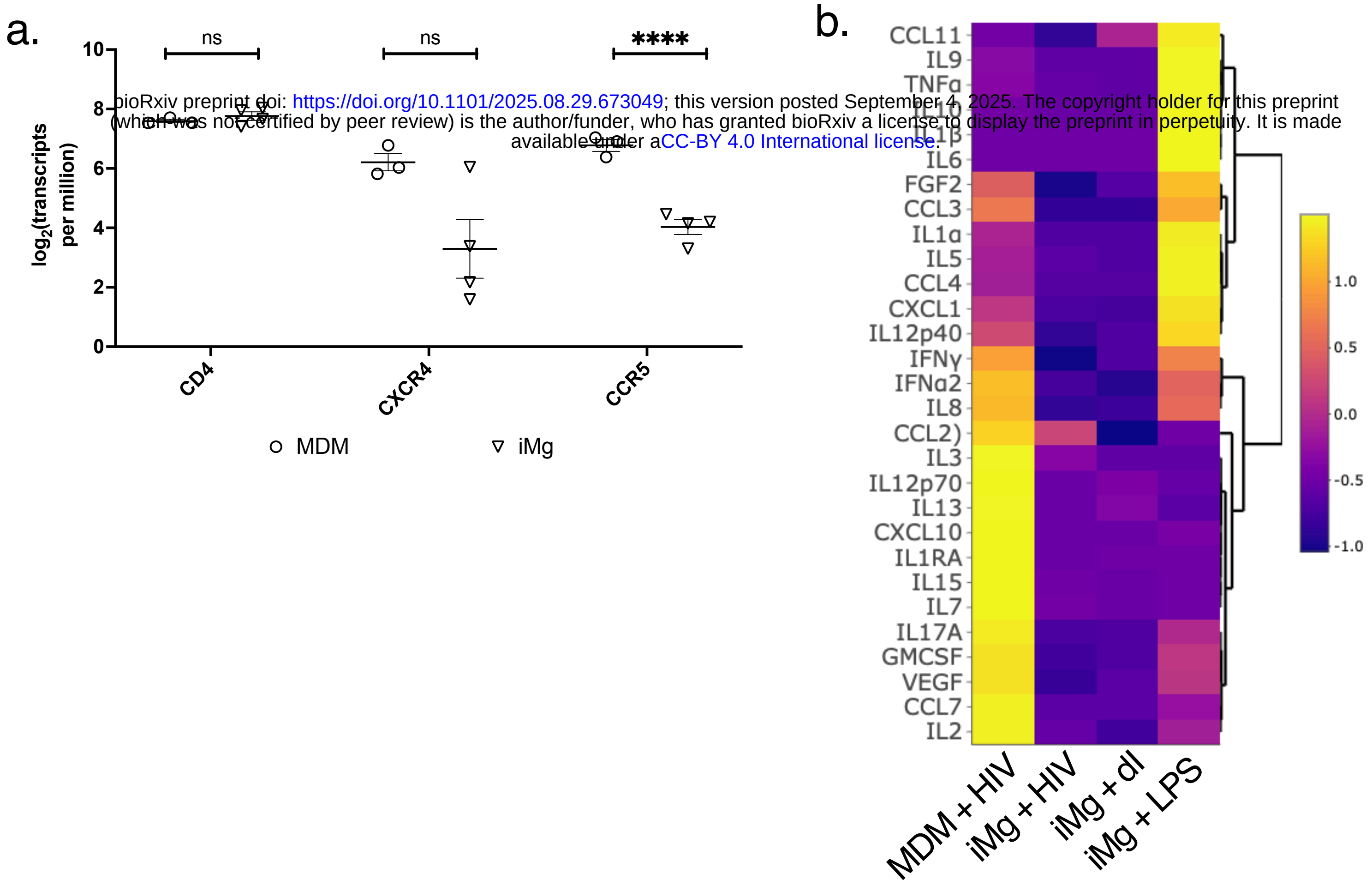

bioRxiv preprint doi: https://doi.org/10.1101/2025.08.29.673049; this version posted September 4, 2025. The copyright holder for this preprint (which was not certified by peer review) is the author/funder, who has granted bioRxiv a license to display the preprint in perpetuity. It is made available under aCC-BY 4.0 International license.

a.

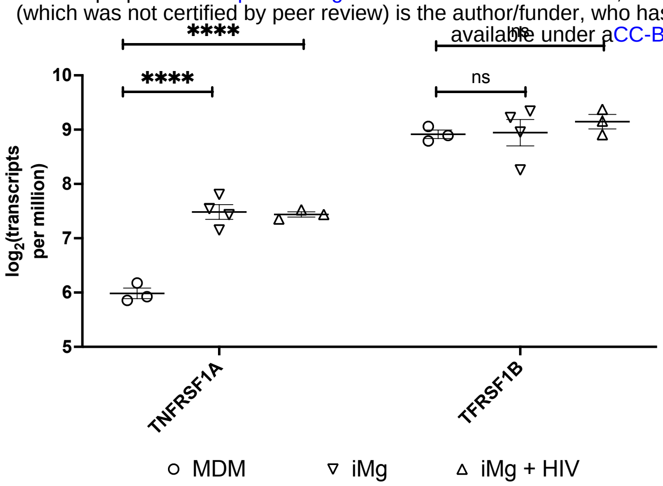

bioRxiv preprint doi: https://doi.org/10.1101/2025.08.29.673049; this version posted September 4, 2025. The copyright holder for this preprint (which was not certified by peer review) is the author/funder, who has granted bioRxiv a license to display the preprint in perpetuity. It is made available under aCC-BY 4.0 International license.

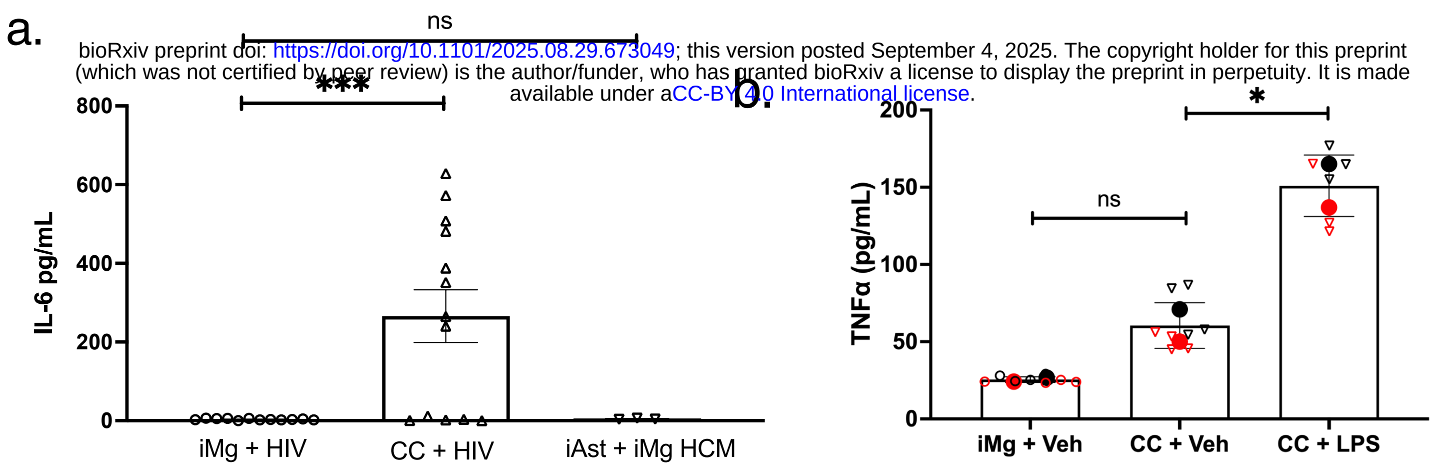

bioRxiv preprint doi: https://doi.org/10.1101/2025.08.29.673049; this version posted September 4, 2025. The copyright holder for this preprint (which was not certified by peer review) is the author/funder, who has granted bioRxiv a license to display the preprint in perpetuity. It is made available under aCC-BY 4.0 International license.

a.

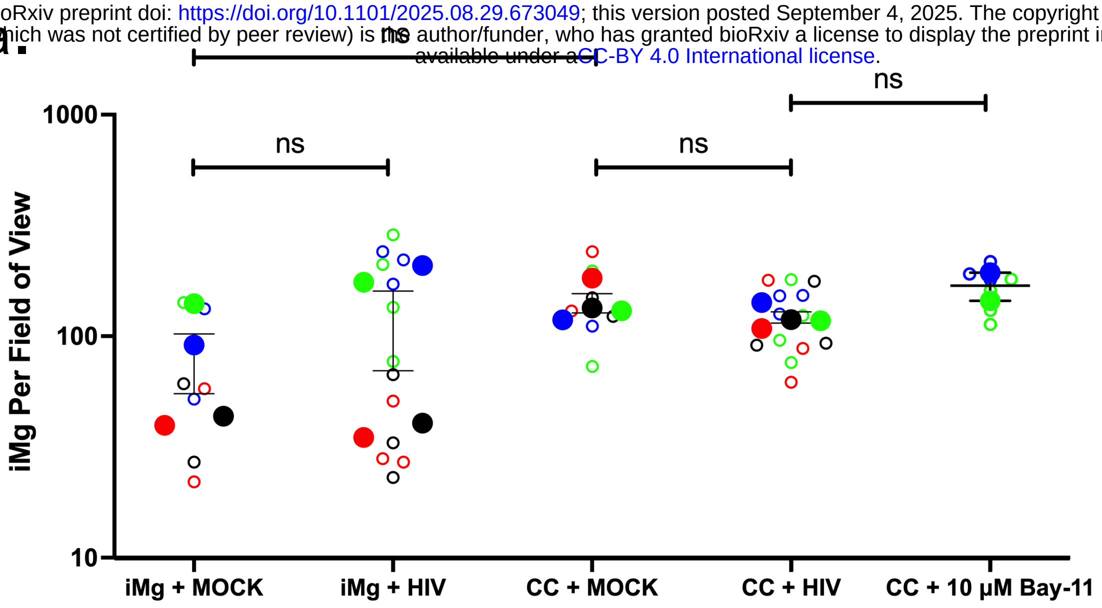

bioRxiv preprint doi: https://doi.org/10.1101/2025.08.29.673049; this version posted September 4, 2025. The copyright holder for this preprint (which was not certified by peer review) is the author/funder, who has granted bioRxiv a license to display the preprint in perpetuity. It is made available under aCC-BY 4.0 International license.

a.

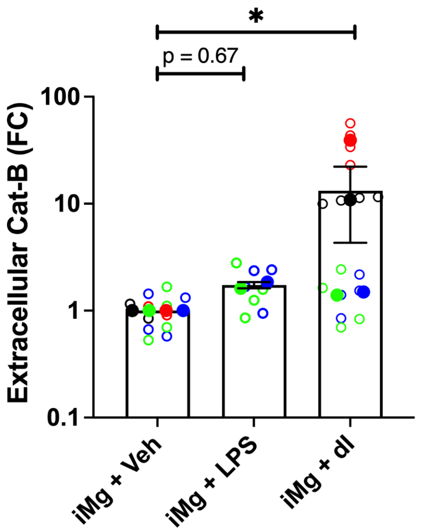

Supplement: Supplement 1 [file NIHPP2025.08.29.673049v2-supplement-1.pdf]
